# The Spatiotemporal Trend of City Parks in Mainland China between 1981 and 2014: Implications for the Promotion of Leisure Time Physical Activity and Planning

**DOI:** 10.3390/ijerph14101150

**Published:** 2017-09-29

**Authors:** Kai Wang, Jianjun Liu

**Affiliations:** College of Landscape Architecture and Arts, Northwest A&F University, Yangling 712100, China; ljj@nwsuaf.edu.cn

**Keywords:** city parks, physical activity, temporal trend, spatial disparity, planning, China

## Abstract

City parks, important environments built for physical activity, play critical roles in preventing chronic diseases and promoting public health. We used five commonly used park indicators to investigate the spatiotemporal trend of city parks in mainland China between 1981 and 2014 at three scales: national, provincial and city class. City parks in China increased significantly with a turning point occurring around the year 2000. Up until the end of 2014, there were 13,074 city parks totaling 367,962 ha with 0.29 parks per 10,000 residents, 8.26 m^2^ of park per capita and 2.00% of parkland as a percentage of urban area. However, there is still a large gap compared to the established American and Japanese city park systems, and only 5.4% of people aged above 20 access city parks for physical activity. The low number of parks per 10,000 residents brings up the issue of the accessibility to physical activity areas that public parks provide. The concern of spatial disparity, also apparent for all five city park indicators, differed strongly at provincial and city class scales. The southern and eastern coastal provinces of Guangdong, Fujian, Zhejiang and Shandong have abundant city park resources. At the scale of the city classes, mega-city II had the highest of the three ratio indicators and the large city class had the lowest. On one hand, the leading province Guangdong and its mega-cities Shenzhen and Dongguan had park indicators comparable to the United States and Japan. On the other hand, there were still five cities with no city parks and many cities with extremely low park indicators. In China, few cities have realized the importance of city parks for the promotion of leisure time physical activity. It is urgent that state and city park laws or guidelines are passed that can serve as baselines for planning a park system and determining a minimum standard for city parks with free, accessible and safe physical activity areas and sports facilities.

## 1. Introduction

City parks are important environments built for leisure time physical activity that plays a key role in meeting recommended physical activity levels in modern lifestyles [1]. Insufficient physical activity and sedentary behavior contribute to a range of chronic diseases including depression, heart disease, hypertension, type 2 diabetes and obesity [2,3]. In China, the prevalence of chronic disease deaths has been reported as high as 87% of deaths. The rate of hypertension and diabetes among Chinese adults rose to 25.2% and 9.7% in 2012, while only 18.1% of adults engage in regular physical activity [4]. The 2014 Chinese Physical Activity Survey Report stated that lack of physical activity areas is the third most important factor that leads to 10% of physical inactivity for people aged above 20 behind lack of time (30.6%) and lack of interest (11.6%) [5]. The provision of accessible, well-equipped and safe city parks has been shown to be an effective strategy to enhance residents’ physical activity [1,6,7]. The latest systematic review of observational park-based physical activity studies shows that 31% to 85% of park users (median = 55%) engage in moderate-to-vigorous physical activity (MVPA) [8].

In addition to the public health perspective, city parks provide a wide variety of services and benefits for urban residents from an economic perspective [9,10], an environmental conservation perspective [11,12] and social benefits perspective [13,14,15]. The history of modern city parks dates from the American parks and recreation movement of the mid-19th century [16]. New York City’s Central Park was established in 1873 and is the first real public park in the United States. Besides natural and manmade beauty, it provides three baseball fields, two basketball courts, 21 playgrounds and many other physical activity facilities to more than 25 million visitors each year [17]. In the United States, there are more than 108,000 public park facilities and 65,000 indoor facilities affiliated to more than 9000 local park and recreation departments and organizations [18]. City parks have been recognized as a health service and part of the healthcare system by the Trust for Public Land, America’s largest national nonprofit organization working to create and improve neighborhood parks. The Center for City Park Excellence in the Trust for Public Land has published reports on city park data annually since 2010. The latest 2017 City Park Facts Report covers the 100 most populous cities, providing detailed data that include park units, parkland, accessibility, facilities and spending to elucidate the realities of urban park and recreation systems [19]. The data and other information are further integrated and used to populate the ParkScore rating system to help improve park systems and identify the priority of residents’ needs for parks. Furthermore, ParkScore results can be applied to other public health studies [20,21] and planning projects such as New York City Playgrounds Program. Reliable data that reflect the status of an urban park and recreation system play an important role in the continual growth and progress of city parks [22,23,24,25,26]. These data underpin a successful park and recreation master plan, e.g., City of Fort Collins Parks and Recreation Policy Plan, Northern Manhattan Parks 2030 Master Plan, etc.

City parks account for a small part of the Chinese urban green space. Chinese cities often have much green area but little parkland. For example, at the end of 2014 Shanghai had 125,741 ha of green space but only 2301 ha of parkland [27]. Compared with other urban green spaces (e.g., green land for production, protected green space and roadway green space), city parks can provide versatile facilities and services to meet public needs, and have a direct and significant effect on human health and wellbeing [1,20]. Many studies in China have focused on the spatial and temporal trends of public parks within city limits, such as Beijing [28,29,30], Shanghai [31,32], Xi’an [33] and Shenzhen [34]. Others include small-scale accessibility analyses [35], post-occupancy evaluation [36] and economic benefits [10]. There have, nevertheless, been few studies conducted on the long-term trend of Chinese city parks on a large scale.

The main goal of this study is to investigate the spatiotemporal trend of city parks in mainland China (hereafter “China”) over the last three decades when its economy and society have rapidly and massively changed. Specifically, the study has two objectives: (1) to detect the temporal trend of city parks on a national level; and (2) to characterize the spatial disparities on a provincial and city class scale. In addition, an effort was made to find out the important issues regarding park-based physical activity and planning. The findings of this study will be useful to understand the city park trends in China which contribute to the promotion of park-based physical activity and city park planning, and provide valuable information for relevant public health and sustainability research. China’s experience will help many other emerging and developing countries that are seeking solutions for improving city parks and public health.

## 2. Methods

### 2.1. Study Sites

City parks were investigated at three scales: national, provincial and city class. Up until the end of 2014, China had 31 provincial-level administrative units, including 27 provinces and four direct-controlled municipalities. The province-scale analysis contained only the 27 provinces (see Table 1). There were 653 cities in all administrative categories. This study used 650 cities as study sites, excluding three cities without reliable data, but including the aforementioned four direct-controlled municipalities, 15 vice-provincial cities, 272 prefecture-level cities and 359 county-level cities. The 650 cities were regrouped into five classes using the latest population-based city classifications released by the Chinese State Council (see Table 2).

### 2.2. City Park Data

All city park data between 1981 and 2014 were obtained from the 2014 China Urban Construction Statistical Yearbook [27]. This Yearbook was compiled using statistical data provided by urban construction authorities of provincial-level administrative units. The city park data used in the Yearbook were reported by the local parks and recreation agencies responsible for the planning, construction and management of city parks. For example, the city park data of Shanghai were reported by the Shanghai Municipal Administration of Afforestation & City Appearance, and can be visited online [37]. The concept of a city park in this paper complies with the definition in the Yearbook. It refers to “places open to the public for the purpose of tourism, appreciation, relaxation, and undertaking scientific, cultural and recreational activities”. Specifically, it includes general parks, theme parks and belt parks. General parks are found in most city systems to ensure that all users’ recreation needs and interests are addressed and included. This type of park, that normally has no less than 10 ha, provides as many recreation and support services as possible and attract users of all ages. Theme parks, also called special use parks, are created to meet the needs of a specific user group, e.g., children’s parks, memorial parks, botanic gardens, etc. These facilities have various sizes depending on the demand and type of layout. Belt parks are linear parks, or environmental corridors, that can be located along streets, preserved city walls (e.g., Xi’an City Wall) waterfronts, or other available public land.

### 2.3. Park Indicators Selection and Data Analysis

Based on a literature review [19,20,21,22,23,38], five indicators were selected to describe city parks: parks (number of city parks), park area (total area of city parks), parks per 10,000 residents (the ratio of number of city parks to urban population in 10,000 persons), park area per capita (the ratio of park area to urban population) and parkland as a percentage of urban area (parkland percentage, the ratio of park area to urban area). Parks and park area are absolute indicators that describe the basic statistical information of city parks. The last three ratio indicators can be used to make domestic and international comparisons.

Number of parks, park area, urban population and urban area data were acquired from the Yearbook. These data were entered into Microsoft Office Excel 2007 to calculate parks per 10,000 residents, park area per capita and parkland percentage data. The five indicators were then analyzed using Microsoft Office Excel 2007 to describe the temporal trend of city parks on a national level and the spatial disparities at the city class scale. ArcMap 10.1 (Esri, Redlands, CA, USA) was used to map the spatial disparities at the provincial scale.

## 3. Results

### 3.1. Temporal Trend of City Parks on the National Level

**Figure 1:**
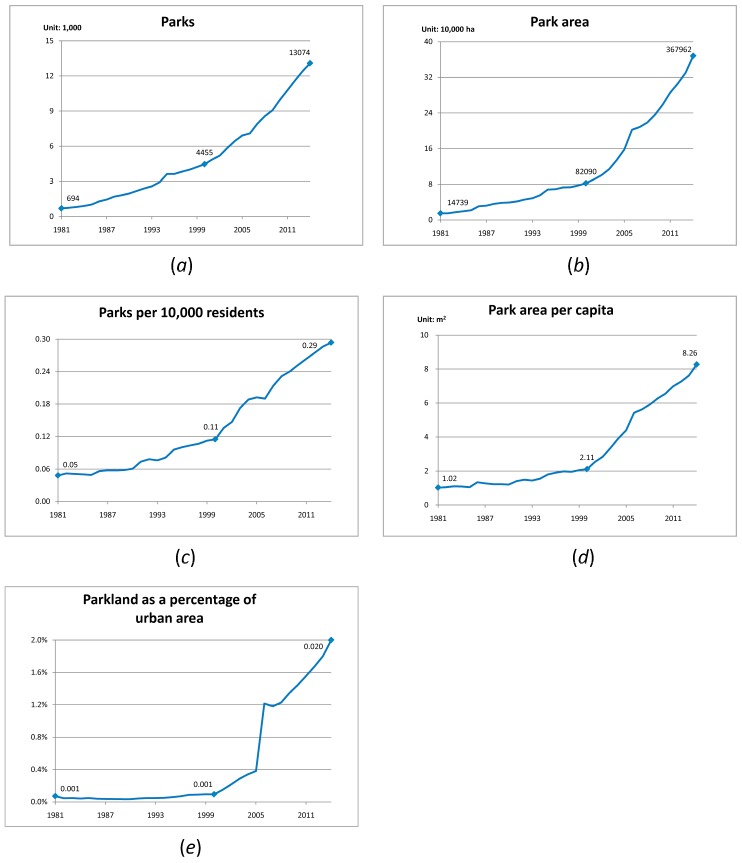
City park statistics in China between 1981 and 2014: (**a**) Parks; (**b**) Park area; (**c**) Parks per 10,000 residents; (**d**) Park area per capita; (**e**) Parkland as a percentage of urban area.

Five indicators (parks, park area, parks per 10,000 residents, park area per capita and parkland percentage) increased significantly between 1981 and 2014 (Figure 1). This 34-year period can be split into two parts with the turning point at the year 2000 based on the difference in growth rate. Before 2000 the growth rate was very low while afterwards it accelerated. In 2014, there were 13,074 parks and park area was 367,962 ha; respectively19 and 25 times more than those in 1981. Parks per 10,000 residents, park area per capita and parkland percentage increased to 0.29, 8.26 m^2^ and 2.00% respectively, i.e., 6, 7 and 28 times more than those in 1981. The growth rate in parkland percentage was the greatest while parks per 10,000 residents was the lowest.

### 3.2. Spatial Characteristics of City Parks at the Provincial Scale

**Figure 2:**
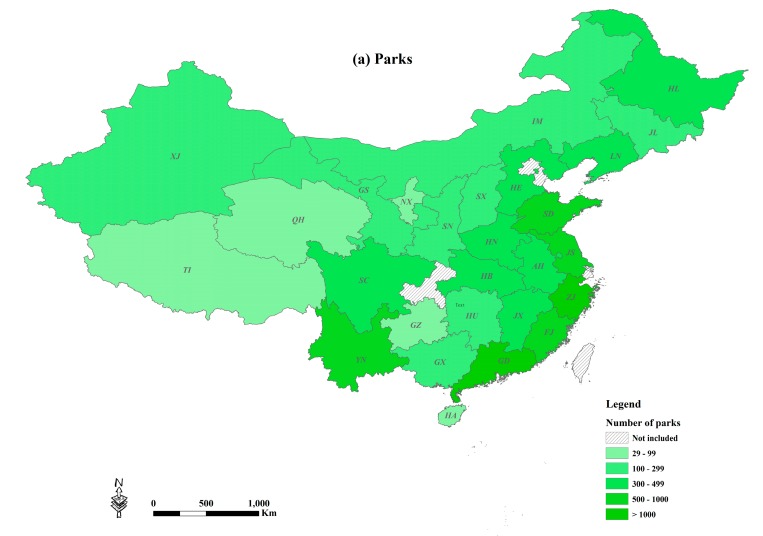

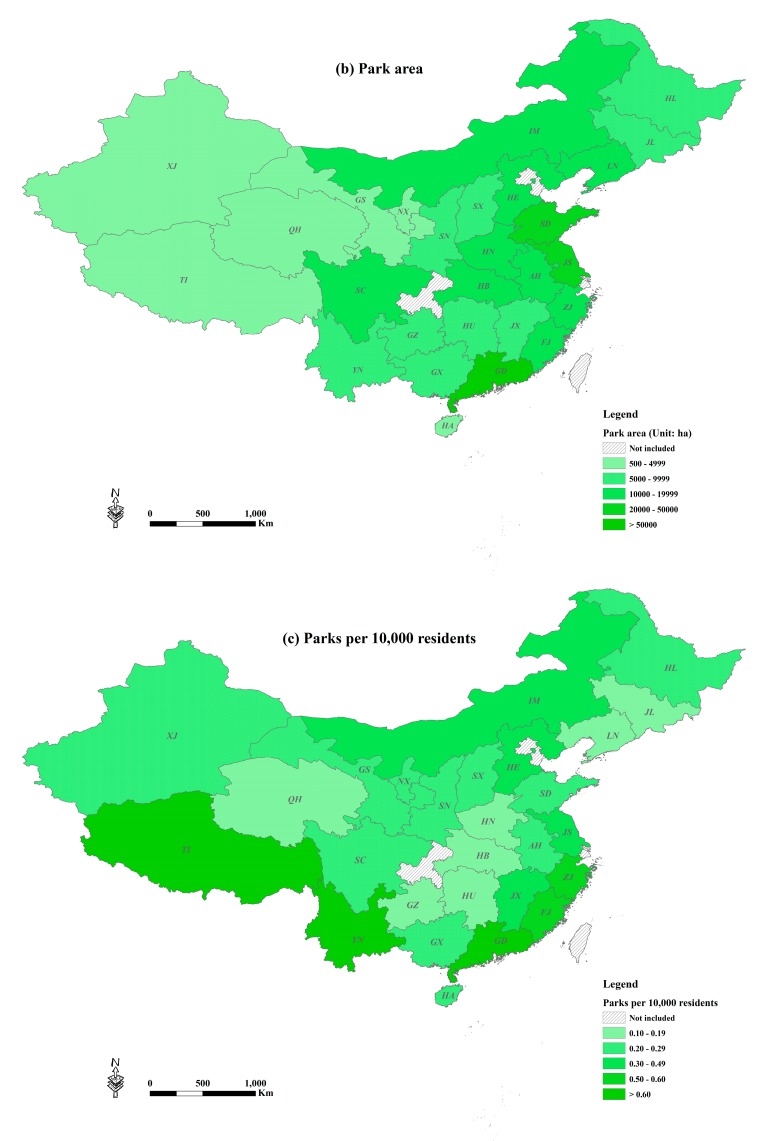

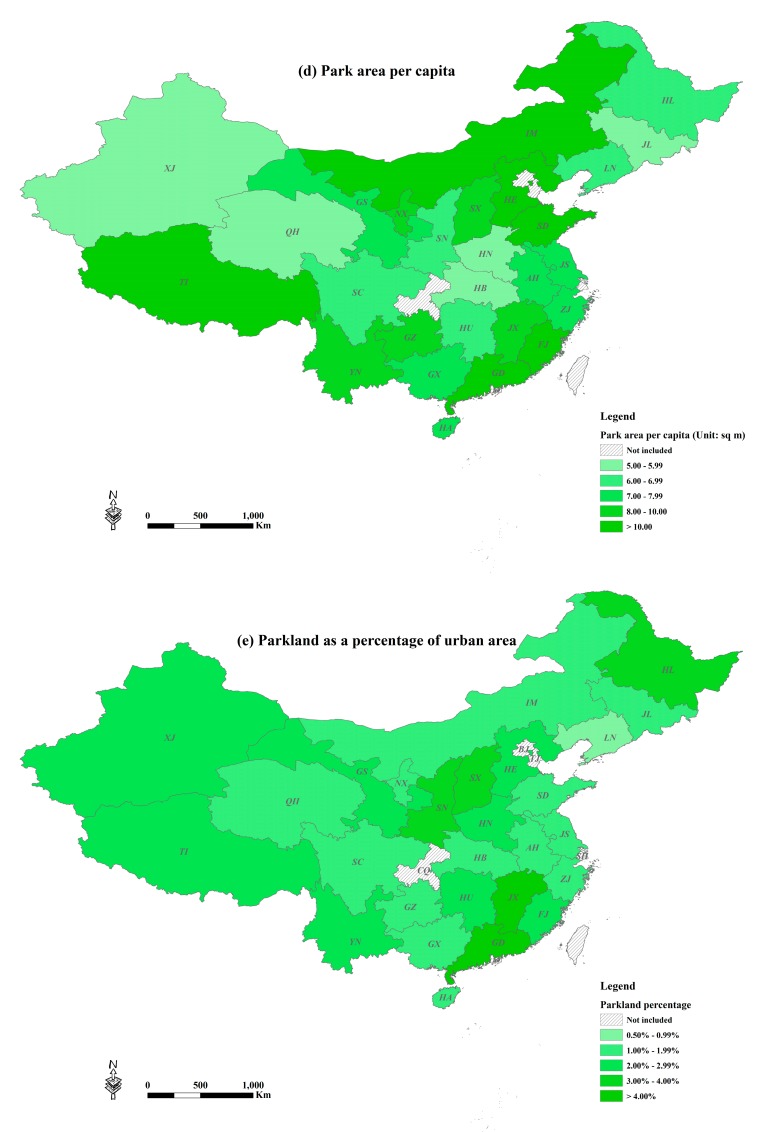
City park statistics in the 27 provinces in China: (**a**) Parks; (**b**) Park area; (**c**) Parks per 10,000 residents; (**d**) Park area per capita; (**e**) Parkland as a percentage of urban area. AH: Anhui, FJ: Fujian, GD: Guangdong, GS: Gansu, GX: Guangxi, GZ: Guizhou, HA: Hainan, HB: Hubei, HE: Hebei, HL: Heilongjiang, HN: Henan, HU: Hunan, IM: Inner Mongolia, JL: Jilin, JS: Jiangsu, JX: Jiangxi, LN: Liaoning, NX: Ningxia, QH: Qinghai, SC: Sichuan, SD: Shandong, SN: Shaanxi, SX: Shanxi, TI: Tibet, XJ: Xinjiang, YN: Yunnan and ZJ: Zhejiang.

All five indicators were mapped at the provincial level (Figure 2). Guangdong had the most and Qinghai the least number of parks (respectively 3408 and 29) with Henan at the median with 306 parks. Guangdong had the most and Tibet the least park area (70,151 ha and 723 ha) with a median of 9555 ha in Hunan. Four provinces had both more than 500 parks and 15,000 ha of park area (Guangdong, Zhejiang, Jiangsu and Shandong). In contrast, there were four provinces with both less than 100 parks and 5000 ha of park area (Ningxia, Hainan, Qinghai and Tibet).

Every 10,000 residents had 0.88, 0.24 and 0.10 parks in Tibet, Shanxi and Guizhou respectively, representing the maximum, median and minimum areas. Urban residents in Inner Mongolia had the largest average park area (13.84 m^2^ per capita). Henan had the smallest park area per capita with each resident getting access to about 5.00 m^2^. Hainan had the median park area with 7.85 m^2^ per capita. Parkland percentage in Guangdong, Sichuan and Liaoning was the highest (4.12%), median (1.92%) and lowest (0.98%) respectively. The national average on the three ratio indicators (parks per 10,000 residents, per capita park area and parkland percentage) was 0.29, 8.26 m^2^ and 2.00% respectively. Four provinces had greater values of these indicators (Guangdong, Hebei, Jiangxi and Tibet).

There were, however, nine provinces lower than the national average (Guizhou, Hainan, Guangxi, Anhui, Qinghai, Liaoning, Sichuan, Hubei and Jilin).

### 3.3. City Park Change at the City Class Level

First of all, the overall change at the city class level was described and compared. Secondly, each city class was analyzed, and the analysis on mega-city I and II was made separately but large city, medium- and small-sized city were combined because of their numbers of cities. Finally, the top ten cities in the five park indicators were reported.

Combined, mega-city I and II with4179 parks and 112,973 ha of park area constitute approximately 32% and 31% of the total number and area of parks. The large city category comprised 41% and 42% in park number and area, and the combined medium- and small-sized city category covered the remaining 27% in both indicators. Of the other three ratio indicators, the mega-city II had higher values than the national average, and the parks per 10,000 residents and park percentage were the highest indicators in all five city classes. In contrast, indicators in the large city class were all lower than the national average. Mega-city I had two indicators lower than the national average; parks per 10,000 residents was the lowest. The medium- and small-sized city classes had one and two indicators higher than the national average, and the park area per capita of the small-sized city was the highest in all five (see Table 3).

There are seven cities in the mega-city I class (see Table 4), including the four direct-controlled municipalities (Beijing, Tianjin, Shanghai and Chongqing) and the other three cities (Wuhan (HB), Guangzhou (GD) and Shenzhen (GD)). In this class, Shenzhen was highest in all indicators except park area (Beijing had the largest park area). Shanghai had the lowest number of parks per 10,000 residents, park area per capita and parkland percentage. Compared with the national average of these three indicators, Shenzhen was greater and Tianjin and Shanghai were lower.

Ten cities are included in the mega-city II class (Shenyang (LN), Harbin (HL), Nanjing (JS), Hangzhou (ZJ), Zhengzhou (HN), Shantou (GD), Foshan (GD), Dongguan (GD), Chengdu (SC) and Xi’an (SN); see Table 5). Dongguan had the greatest values in all the five indicators. Shantou had the lowest values in parks, park area and parks per 10,000 residents, and Zhengzhou and Shenyang had the lowest values in park area per capita and parkland percentage respectively. Dongguan and Foshan were higher than the national average while Shenyang and Shantou were lower.

Large cities, in the combined large, medium- and small-sized city class, were superior to medium- and small-sized cities in parks and park area, but small-sized cities had better ratio indicators (see Table 6). Significant skewness can be observed in all five indicators across the three classes. A large portion of cities clustered at the lower end and many clearly demonstrated a serious deficiency of city parks (see Table 7). In total, there were 379 cities with fewer than 10 parks, 156 cities with less than 100 ha of park area, 80 cities with fewer than 0.1 parks per 10,000 residents, 101 cities with less than 3 m^2^ of park per capita and 176 cities with less than 1% parkland.

Table 8 lists the top ten cities in the five park indicators. The top ten cities for parks included four from the mega-city I, three from the mega-city II and three from the large city classes. Their numbers ranged from 1210 to 164. With the exception of small-sized cities, the other four classes appear in the top ten cities for park area (four from mega-city I, two from mega-city II, three from large city and one from medium-sized city). The range was from 28,798 to 3313 ha. The mega-city I and II classes barely appeared in the top ten cities among ratio indicators, except Dongguan in the parks per 10,000 residents. The other nine cities that appeared in the top ten of parks per 10,000 residents were one from the large city, two from the medium-sized city and six from the small-sized city classes, ranging from 3.60 to 1.45 parks per 10,000 residents. The results were similar in the park area per capita and the parkland percentage. The small-sized city class covered seven of ten in both park area per capita and parkland percentage while one from the large city and two from the medium-sized city completed the list. These ranged from 66.99 to 26.13 ha park area per capita, and from 23.22% to 11.16% parkland percentage respectively.

## 4. Discussion

City parks are valuable resources for leisure time physical activity. The number of city parks in mainland China increased significantly between 1981 and 2014. The turning point occurred around the year 2000, especially for the ratio indicators. However, numbers are still small compared with the established American and Japanese city park systems. Up until the end of 2014, China (650 cities in this study) had 13,074 city parks with an area of 367,962 ha. It averaged 0.29 parks per 10,000 residents, 8.26 m^2^ park per capita and 2.00% of parkland percentage. The 100 most populous cities in the United States had 21,980 parks totaling 819,569 ha in 2015. It averaged 3.48 parks per 10,000 residents, 129.79 m^2^ of park per capita and 17.68% of parkland percentage [19]. In 2014, there were 105,744 city parks totaling 122,839 ha in Japan with 8.78 parks per 10,000 residents, 10.2 m^2^ park per capita and 1.21% parkland percentage [39]. Since the Japanese land area is just less than 4% of China, and its population density is double, it is unattainable to have as much park area as the United States. To provide accessibility and fulfill the level of service, the Japanese solution is to develop many city parks within a limited area. On one hand, one observational study reported that more than 50% of Chinese park users took part in MVPA [40]. On the other hand, only 5.4% of people aged above 20 have access to no or low-cost city parks for physical activity [5]. The conflict suggests that more city parks with sports facilities should be planned and constructed to meet public needs on free, accessible and safe physical activity areas. Likewise, the National Fitness Program (2016–2020) stresses the construction of public sports facilities, especially making full use of underutilized city parks and remnant or obsolete urban land [41].

All five city park indicators differed across spatial scales. At the provincial level, the general trend is that the East of China was superior to the West, and the South was better than the North. The southern and eastern coastal provinces of Guangdong, Fujian, Zhejiang and Shandong had abundant city park resources. City parks were scarce in the northeastern Jilin, and in the northwestern Shaanxi, Qinghai and Xinjiang. Other provinces that had poor indicators included the middle Henan, southwestern Sichuan and southern Hunan. Southwestern Yunnan has relatively high city park indicators.

At the scale of the city classes, mega-city I had the lowest number of parks per 10,000 residents, but the highest parkland percentage. This indicated that there were many large city parks in this class, and a lack of neighborhood and pocket parks with small areas. This is likely to result in an issue of inaccessibility. An example of this is the study carried out by Yuan and Xu [42] in which only about 30% of the city area and 43% of the population were within a1000 m service radius of six downtown districts in Beijing. The six downtown districts had 213 city parks with an area of 7043 ha, and the average park area was 33 ha. In this study, Beijing as a whole had as much as an average of 102 ha of park area. Mega-city II was at the top of the three ratio indicators. In the large, medium- and small-sized city, the parkland percentage was lower than the national average, which implies that their growth in city park area was slower than urban sprawl. Since only five percent of the urban population resided in small-sized cities, its parks per 10,000 residents and park area per capita were higher than the national average. The 149 small-sized cities had only 983 parks and 21,947 ha of park area. One hundred and nineteen cities had less than ten parks and 62 cities had less than 100 ha of park area. More parks are needed to provide recreation and physical activity opportunities for local residents in these cities, where illegal gambling activities and recreational drug use are quickly rising [43].

In addition to divergence among city classes, it can also be seen within a province. For example, five of 17 mega-cities were in the highly rated province of Guangdong. The ratio indicators of Shenzhen, Dongguan and Foshan were above the national average, but those in Shantou were below.

China has achieved unprecedented growth in economic development and urbanization for the last 30 years. Unfortunately, it also became the victim of poorly planned growth, and its citizens are suffering some consequences, especially urban isolation and environmental pollution [44]. Widespread, uncontrolled smog is one strong indicator. Historically, public city parks were established to tackle urban problems accompanying large-scale industrialization in the early 19th century [16]. Some developed countries, such as the United States and Japan, have established successful city park systems. This raises the question of whether China could copy their success in city parks. Studying existing successful systems would benefit city park planning and policy. However, differences in national history, urban development, the relationship between population and land, and the residents’ perception of public spaces will determine how China will find its solution to the challenges of establishing and maintaining city park systems. As the world’s largest developing country, the study of China’s city parks will broaden international knowledge and be useful for other densely populated developing countries.

Several limitations of this study should be noted. First, the temporal trend of city parks was only identified on a national level. Due to the limitation of the data source, the trend at the provincial scale was not included. However, the trend in Guangdong should be distinct from that in Zhejiang, even though both of them had abundant city park resources in 2014. Second, intra-province spatial disparities were not analyzed. The park-poor province may have a couple of cities with high park indicators, and vice versa. Third, a few temples or historic sites were treated as city parks by some local parks and recreation agencies, e.g., Ba Xian An Monastery (Temple of the Eight Immortals) and Da Xingshan Temple in the city of Xi’an. The degree of bias is yet unknown. Finally, besides the five selected park indicators, there are a couple of key indicators that have a significant effect on the quality of city parks from the perspective of promoting leisure time physical activity which are not included, e.g., the percentage of urban population with walkable park access, total park spending per resident and park playgrounds per 10,000 residents. Future research is recommended in the following areas: the accessibility and equity of city parks, park auditing based on remote sensing and crowdsourcing, and park usage and physical activity.

## 5. Conclusions

According to the National Fitness Program (2016–2020), by 2020, a significant growth is expected in the number of people who are physically active, and people’s physical fitness will be steadily improved [41]. As valuable access to physical activity opportunities, city parks in mainland China have increased significantly between 1981 and 2014, but there are still few for residents’ physical activity. Compared with the American and Japanese city park systems, the large difference in parks per 10,000 residents may result in an accessibility issue for public park services. The concern of spatial disparity is also apparent across the scales of province, city class and individual cities. On one hand, the leading province Guangdong and its mega-cities Shenzhen and Dongguan have park indicators comparable to the United States and Japan. Guangdong has built more than 12,000 km of greenways, and newly completed 367 community sports parks by the end of 2015. On the other hand, there are still five cities with no city parks and many cities with extremely low park indicators. According to the ongoing prioritized urbanization policy, the disparities will be expected to grow if no counter measures are taken.

Although urban green space system planning has had a positive effect on the growth of city parks, specific city park planning should be conducted, particularly to address the park-deficit in mega-cities and populated large cities, such as Shanghai, Tianjin and Shantou. Parks, recreation and open space master plans have long been proven to be an effective tool in the United States. Few Chinese cities have realized the importance of city park planning. Only Wuxi and Shenzhen published their own city park master plan in 2013, and Guangzhou called for a bid in 2016. Meanwhile, it is urgent to pass city park laws or guidelines that can serve as an appropriate baseline for planning a park system or determining the minimum standard for a city park.

City parks as pivotal public spaces and vital green infrastructure are of strategic importance in the pursuit of the sustainable city and the wellbeing of urban dwellers. However, little progress has been made in China’s city park studies. This is partly because the spatial data of city parks and other relevant data such as population grids, road networks and population health are not available or not openly accessible. In addition, more financial support should be granted since both the National Natural Science Foundation of China and the National Planning Office of Philosophy and Social Science have approved no more than 22 research project applications on city parks in the past 30 years.

## Figures and Tables

**Table 1:** Twenty-seven provinces in China.

Province	Abbreviation	No. of Cities	Population (10,000 Persons)
Hebei	HE	31	1628.70
Shanxi	SX	22	1084.45
Inner Mongolia	IM	20	873.46
Liaoning	LN	31	2274.80
Jilin	JL	28	1154.98
Heilongjiang	HL	29	1378.30
Jiangsu	JS	36	2978.17
Zhejiang	ZJ	31	2028.05
Anhui	AH	22	1432.38
Fujian	FJ	22	1134.15
Jiangxi	JX	21	987.80
Shandong	SD	45	3038.71
Henan	HN	38	2400.63
Hubei	HB	36	1879.96
Hunan	HU	29	1457.90
Guangdong	GD	42	5109.36
Guangxi	GX	21	991.08
Hainan	HA	8	264.14
Sichuan	SC	32	1971.35
Guizhou	GZ	13	632.52
Yunnan	YN	20	828.35
Tibet	TI	3	67.15
Shaanxi	SN	13	881.45
Gansu	GS	16	572.43
Qinghai	QH	5	165.58
Ningxia	NX	7	273.45
Xinjiang	XJ	25	724.09

**Table 2:** Five population-based city classes.

City Class	No. of Cities	Population (10,000 Persons)	Standard
Mega-City I (MC I)	7	9131.28	>ten million inhabitants
Mega-City II (MC II)	10	4525.72	Between five and ten million inhabitants
Large city (LC)	211	19,361.48	Between one and five million inhabitants
Medium-Sized city (MSC)	273	9030.32	Between 0.5 and one million inhabitants
Small-Sized city (SSC)	149	2320.41	<0.5 million inhabitants
Total	650	44,369.21	

**Table 3:** City park statistics in the five city classes in 2014.

City Class	Parks	Park Area (ha)	Parks per 10,000 Residents	Park Area per Capita (m^2^)	Parkland Percentage
MC I	2052	74,217	0.22	8.13	2.29%
MC II	2127	38,756	0.47	8.56	2.68%
LC	5303	156,180	0.27	8.07	1.98%
MSC	2563	75,384	0.28	8.35	1.73%
SSC	983	21,947	0.42	9.46	1.61%
Total	13,074	367,962	0.29	8.26	2.00%

Notes: MC I: mega-city I; MC II: mega-city II; LC: large city; MSC: medium-sized city; SSC: small-sized city.

**Table 4:** City park statistics in the mega-city I in 2014.

City	Parks	Park Area (ha)	Parks per 10,000 Residents	Park Area per Capita (m^2^)	Parkland as a Percentage of Urban Area
Beijing	282	28,798	0.15	15.49	2.36%
Tianjin	94	2124	0.12	2.70	0.89%
Shanghai	161	2301	0.06	0.95	0.36%
Wuhan	74	3110	0.12	4.90	2.14%
Guangzhou	245	5180	0.22	4.69	3.71%
Shenzhen	889	21,953	0.82	20.37	10.99%
Chongqing	307	10,751	0.25	8.65	1.62%

**Table 5:** City park statistics in the mega-city II in 2014.

City	Parks	Park Area (ha)	Parks per 10,000 Residents	Park Area per Capita (m^2^)	Parkland as a Percentage of Urban Area
Shenyang	66	3138	0.13	6.07	0.97%
Harbin	90	1868	0.22	4.48	4.66%
Nanjing	120	6861	0.20	11.27	1.62%
Hangzhou	193	2102	0.50	5.45	2.05%
Zhengzhou	74	2260	0.17	3.54	5.14%
Shantou	28	1198	0.11	4.75	1.97%
Foshan	190	1961	0.94	9.71	2.57%
Dongguan	1210	14,530	2.00	23.97	5.89%
Chengdu	81	2465	0.16	4.92	3.05%
Xi’an	75	2373	0.19	5.96	5.27%

**Table 6:** City park statistics in the large, medium- and small-sized city classes in 2014.

Statistics	Parks	Park Area (ha)	Parks per 10,000 Residents	Park Area per Capita (m^2^)	Parkland as a Percentage of Urban Area
Large city					
Maximum	463	4158	3.04	34.39	14.38%
Minimum	1	3	0.04	0.14	0.04%
Median	15	468	0.21	6.97	1.81%
Medium-Sized city *					
Maximum	73	3778	3.60	66.99	15.65%
Minimum	1	1	0.03	0.07	0.01%
Median	7	192	0.22	7.14	1.63%
Small-Sized city *					
Maximum	56	1393	3.29	44.55	23.22%
Minimum	1	2	0.06	0.19	0.01%
Median	5	117	0.40	8.58	1.95%

* Two cities in MSC and three cities in SSC with no city parks are not included, i.e., Yuanping (SX, MSC), Fuyu (JL, MSC), Changdu (TI, SCC), Yushu (QH, SCC) and Tiemenguan (XJ, SCC).

**Table 7:** The deficiency of city parks in the large, medium- and small-sized cities in 2014.

Park Indicator	LC (211 Cities)	MSC (273 Cities)	SSC (149 Cities)
Parks (<10)	69 (33%)	188 (69%)	122 (82%)
Park area (<100 ha)	20 (9%)	71 (26%)	65 (44%)
Parks per 10,000 residents (<0.1)	31 (15%)	35 (13%)	14 (9%)
Park area per capita (<3 m^2^)	28 (13%)	49 (18%)	24 (16%)
Parkland percentage (<1%)	41 (19%)	88 (32%)	47 (32%)

**Table 8:** The top ten cities in parks, park area, parks per 10,000 residents, park area per capita and parkland percentage in 2014.

City	Province *	City Class	City	Province *	City Class
Parks			Park Area (ha)		
Dongguan	GD	MC II	Beijing	DCM	MC I
Shenzhen	GD	MC I	Shenzhen	GD	MC I
Kunming	YN	LC	Dongguan	GD	MC II
Chongqing	DCM	MC I	Chongqing	DCM	MC I
Beijing	DCM	MC I	Nanjing	JS	MC II
Guangzhou	GD	MC I	Guangzhou	GD	MC I
Hangzhou	ZJ	MC II	Shijiazhuang	HE	LC
Foshan	GD	MC II	Linyi	SD	LC
Suzhou	JS	LC	Zhaoqing	GD	MSC
Zhuhai	GD	LC	Taiyuan	SX	LC
Parks per 10,000 residents			Park area per capita (m^2^)		
Changle	FJ	MSC	Zhaoqing	GD	MSC
Lijiang	YN	SSC	Jinggangshan	JX	SSC
Kunshan	JS	LC	Arxan	IM	SSC
Wuyishan	FJ	SSC	Ulanqab	IM	SSC
Dongguan	GD	MC II	Xigaze	TI	SSC
Suifenhe	HL	SSC	Dongying	SD	LC
Dexing	JX	SSC	Erdos	IM	MSC
Quzhou	ZJ	MSC	Lijiang	YN	SSC
Fenyang	SX	SSC	Zhongwei	NX	SSC
Ruili	YN	SSC	Tumxuk	XJ	SSC
Parkland as a percentage of urban area					
Ulanqab	IM	SSC			
Jinggangshan	JX	SSC			
Sanmenxia	HN	SSC			
Wanyuan	SC	MSC			
Lijiang	YN	SSC			
Lianjiang	GD	LC			
Arxan	IM	SSC			
Xichang	SC	MSC			
Lvliang	SX	SSC			
Heyuan	GD	SSC			

* DCM: Direct-controlled municipality.
